# Dietary differences are reflected on the gut prokaryotic community structure of wild and commercially reared sea bream (*Sparus aurata*)

**DOI:** 10.1002/mbo3.202

**Published:** 2014-07-25

**Authors:** Konstantinos A Kormas, Alexandra Meziti, Eleni Mente, Athanasios Frentzos

**Affiliations:** 1 Department of Ichthyology & Aquatic Environment, School of Agricultural Sciences, University of Thessaly384 46 Volos, Greece; 2 Kefalonia FisheriesLivadi, Lixouri Kefalonia, 28200, Greece

**Keywords:** 16S rRNA, Archaea, Bacteria, gut, pyrosequencing, *Sparus aurata*

## Abstract

We compared the gut prokaryotic communities in wild, organically-, and conventionally reared sea bream (*Sparus aurata*) individuals. Gut microbial communities were identified using tag pyrosequencing of the 16S rRNA genes. There were distinct prokaryotic communities in the three different fish nutritional treatments, with the bacteria dominating over the Archaea. Most of the Bacteria belonged to the Proteobacteria, Firmicutes, Actinobacteria, and Bacteroidetes. The number of bacterial operational taxonomic units (OTUs) was reduced from the wild to the conventionally reared fish, implying a response of the gut microorganisms to the supplied food and possibly alterations in food assimilation. The dominant bacterial OTU in all examined fish was closely related to the genus *Diaphorobacter*. This is the first time that a member of the *β*-Proteobacteria, which dominate in freshwaters, are so important in a marine fish gut. In total the majority of the few Archaea OTUs found, were related to methane metabolism. The inferred physiological roles of the dominant prokaryotes are related to the metabolism of carbohydrates and nitrogenous compounds. This study showed the responsive feature of the sea bream gut prokaryotic communities to their diets and also the differences of the conventional in comparison to the organic and wild sea bream gut microbiota.

## Introduction

Over the last few years, the advent of the – omics technologies has boosted our insights on the structure and function of the complex gastrointestinal tract (GIT) prokaryotic communities. Most of this research has been focused on the human gut microbiome and the gained knowledge has unraveled the unprecedented functions of the microorganisms hosted in our GIT. For example, the general prevailing notion that gut microorganisms are involved in the host's nutritional metabolism and immunity has now been clarified by the elucidated specific metabolic pathways of GIT microorganisms (Li et al. 2008; Verberkmoes et al. 2008), how they associate to the immune system (Kau et al. 2011; Sommer and Bäckhed 2013) or even their involvement in human development (Heijtz et al. 2011). Moreover, it has been shown that GIT seems to be an excellent “evolutionary playground” for horizontal gene transfer among its hosting bacteria, with some of these genes being related to critical for the host metabolic attributes-like virulence, antibiotic resistance, xenobiotic metabolism (Smillie et al. 2011). One of the first steps in the study of the GIT microbial community is to reveal its structural diversity (Eckburg et al. 2005; Marchesi 2010; Huse et al. 2012; Lozupone et al. 2012). By doing so, it is feasible to define its true, that is, resident, from transient members, those that are associated with healthy state of the host or disturbed environmental stressors, like change in diet (e.g., David et al. 2014).

The last few years, the advancements in human gut microbiology have sparked a reinvigorated interest on the study of structural and functional diversity of gut microorganisms for wild and domesticated animals spanning in several major bacterial taxa (e.g., Wong et al. 2013, Zened et al. 2013). Regarding fish, the majority of the available literature on GIT microorganisms refers to the roles of probiotics and immunity (reviewed by Ringø and Gatesoupe 1998; Balcázar et al. 2006; Gómez and Balcázar 2008; Nayak 2010; Ringø et al. 2010). Fish GIT microbial diversity remains considerably understudied (Sullam et al. 2012) and relies mostly on cultivation approaches (e.g., Sivasubramanian and Ravichandran 2012) despite the significant number of fish species among the vertebrates and their global-scale ecological importance (Wong & Rawls, 2012). Finally, and as is the case in most of the investigated microbial habitats, the well-known lower species diversity of Archaea (Smeti et al. 2013) has refrained scientists from including Archaea in the investigations of fish GIT microorganisms (Ni et al. 2014).

The ongoing positive growth trend of the aquaculture industry is expected to continue, reflecting the rising demand for healthy human food products. Therefore, in the past 15 years, there was a rise in demand for seafood that has been farmed according to certified organic standards, notably in European countries (Mente et al. 2011). Organic aquaculture has attracted the attention of consumers as it is reducing health risks with the goals of increasing product quality and environmental performance (Prein et al. 2012). The sector is regulated and has species to species international specific standards, certification procedures, and accreditation bodies. Today, organic salmon and organic trout are the most important organic fish in the market (Bergleiter 2011). The Mediterranean species (sea bream and sea bass) can be compared to organic salmon, but have not yet had the same duration of mainstreaming (Bergleiter 2011). The total European production of sea bass and sea bream was estimated to be 275,000 tons in 2012, but just 1300 tons of this is estimated to have been certified to organic standards nevertheless, it has good expectations of production growth (Prein et al. 2012).

Managing the health and nutrition of cultured aquatic organisms was one of the greatest challenges and opportunities for an expansion of a sustainable production of aquaculture. Current research on human health is focusing on the role of gut microbiota (Flint et al. 2012a, b) and how diet affects the intestinal microbiome (Walker et al., 2011). Studies exist on the effect of the diet on the intestinal microbiota, structure, and morphology of fish (Bakke-McKellep et al. 2007; Dimitroglou et al. 2009; Desai et al. 2012). The future of aquaculture nutrition will benefit from a better understanding of the nutritional strategies and the fish's gut/microbe interactions and gut microorganism's diversity to allow the production of top-quality aquafeeds.

To date very little is known on the diversity and functional role of sea bream's GIT microbial communities. The available data refer only to cultivable bacteria (Floris et al. 2013), which always represent a very small percentage of the existing diversity in any habitat, and the response of the gut bacterial community to the provision of probiotics and prebiotics (Cerezuela et al. 2013). Another study has focused on the effect of diet on the bacterial diversity of the gut content and not the gut tissue itself (Silva et al. 2011). No data exist on the diversity of Archaea associated with the sea bream. Wild gilthead sea bream is mainly carnivorous consuming a variety of prey with molluscs, mussels, crustaceans, and fish as the major dietary groups. The trophic level of the gilthead sea bream is about 3.3–3.5 (www.fishbase.org). The conventionally reared sea bream was fed a commercial diet of 46% protein and 17% fat. The organically reared sea bream was fed an organically produced feed that included sustainable certified fish meal and fish oil (45% protein, 14% fat and no synthetic amino acids) and organic wheat. In this study, we used tag pyrosequencing of the 16S rRNA gene to compare intestinal structural diversity of Bacteria and Archaea on wild, organic, and conventional-reared sea bream (*Sparus aurata*) individuals. The aim of this study was to identify frequently occurring intestinal microorganisms in relation to the nutritional status of the fish.

## Materials and Methods

### Rearing conditions and diets

Gilthead sea bream of ca. 435 g initial average weight were obtained from a commercial fish farm in Greece (Table1). Fish were grown in sea net cages. Similarly to Carter et al. (2012), we used two treatments, organic and conventional, which differed in stocking density, type of feed, and whether antibiotic treatments were given to the fish. The conventionally reared fish – hereafter referred to as “conventional”- were kept in cages at a density of 12 kg m^−3^ while the organically reared fish – hereafter referred to as “organic” – were kept in cages, 500 m away from the conventional cages, at a density of 10 kg m^−3^. Fish were fed the two diets ad libitum the organic and the conventional one, for a period of 12 months. Fish were fed to satiation the organic and the conventional diet respectively, for a period of 12 months.

**Table 1:** Sea bream (*Sparus aurata*) morphometric data and 16S rRNA gene pyrosequencing data of their gut prokaryotes.

	Wild	Organic	Conventional
Length	18.4 ± 0.69	25.2 ± 0.15	24.5 ± 0.47
Weight	168 ± 25.3	464 ± 17.8	409 ± 33.7
HSI (%)	0.42 ± 0.10	0.96 ± 0.07	1.36 ± 0.20
Bacteria reads (average ± SD)	6300 ± 4184.4	9228 ± 4021.4	5091 ± 4260.6
Bacteria OTUs (average ± SD)	164 (65 ± 9.5)	126 (58 ± 14.7)	74 (33 ± 15.0)
Treatment-specific bacteria	133	88	40
Archaea reads	1542	5022	235
Archaea OTUs	9	14	10
Treatment-specific archaea	6	10	7

The temperature ranged from 10.5 to 26.7°C and the dissolved oxygen, measured by a WTW (Weilheim Germany) probe, from 8.1 to 9.6 mg L^−1^. pH ranged from 8.0 to 8.1 throughout the growth cycle. Organically produced feed that included sustainable certified fish meal, fish oil, and certified organic grains, free of any genetically modified organisms or synthetic amino acids was given daily to the organically farmed sea bream ad libitum by hand allowing natural feeding behavior. The organic diet quality is strictly controlled and certified organic by a certified body according to the national and EU regulations. The fish were weighed at monthly intervals, and the feeding level was adjusted as a percentage of the fish total biomass according to the manufacturer (to keep the feeding rate at a constant percentage of body weight). The conventional fish were fed a commercial feed that included fish meal, fish oil, soy bean meal, wheat meal, and corn gluten daily ad libitum by hand. Wild gilthead sea bream were obtained from local fishermen. Since we were able to have only three wild individuals, we analyzed three individuals from each treatment in order to have comparable sample size.

### Sample collection and analysis

At the end of the growth cycle, a total of 10 fishes from both treatments were randomly sampled from the population for further analysis. Fishes were sacrificed by emersion on ice and wet weight was measured. Tissues samples (liver and gut) were dissected out, weighed, and frozen in −80°C freezers until further analysis. Samples of wild fish of similar weights were taken for comparisons.

The hepatosomatic index (HSI) was calculated as HSI = (*W*_Liver_/BW) × 100, where *W*_Liver_ is the liver weight (g) of the sampled fish and BW is the weight (g) of the sampled fish. Mean values with their standard error (SE) are presented in this paper. Homogeneity of variance was confirmed using Levene's test. Multivariate analysis of variance (MANOVA) was used to analyze the relationship between gut microorganisms and dietary condition. Significance was accepted at 5% or less. All statistical analyses were carried out using the PAST software (Hammer et al. 2001).

### Fish gut sampling and DNA extraction

Three healthy looking animals, that is, no visual signs of disease or parasites on the skin and internal organs, from each treatment, that is, wild (W), organic (O), and conventional (C), were dissected using sterile lancets and forceps. The gut was transferred in sterile particle-free (<0.2 *μ*m) sea water (SPFSW). The gut's containing material was extruded by mechanical force with a forceps, as we targeted the resident gut microorganisms and not the ones associated with the ingested food. The evacuated gut was thoroughly rinsed four to five times in SPFSW and was transferred in a sterile plastic vial which was kept at −80°C until DNA extraction. The time lapse from fish sampling to freezing the gut at −80°C never exceeded 5 h, during which all biological material was held in ice. DNA extraction from each individual gut was performed using the PowerMax Soil DNA Isolation kit (MoBio, Carlsbad, CA, USA) according to manufacturer's protocol. Regarding Archaea, only one sample from each treatment had amplifiable DNA.

### Pyrosequencing and data analysis

Tag pyrosequencing of the V1–V3 region of the 16S rRNA gene was amplified by using the primer pair 27F (5′-AGRGTTTGATCMTGGCTCAG-3′) and 519R (5′-GTNTTACNGCGGCKGCTG-3′) for bacteria and arch344F (5′-ACGGGGYGCAGCAGGCGCGA-3′) and arch915R (5′-GTGCTCCCCCGCCAATTCCT-3′) for Archaea were performed as described in Dowd et al. (2008). In brief, a one-step 30 cycle PCR was applied using HotStarTaq Plus Master Mix Kit (Qiagen, Valencia, CA). PCR conditions included: 94°C for 3 min, then followed by 28 cycles of 94°C for 30 sec; 53°C for 40 sec and 72°C for 1 min; and a final elongation step at 72°C for 5 min. Following PCR, all amplicon products (ca. 450 bp) from different samples were mixed in equal concentrations and purified using Agencourt Ampure beads (Agencourt Bioscience Corporation, Beverly, MA, USA), but nine distinct tags (multiplex identifiers, MIDs) were used for the nine samples. Samples were sequenced utilizing Roche (Barnford, CT, USA) 454 FLX titanium instruments and reagents after following manufacturer's guidelines at the MRDNA Ltd. (Shallowater, TX) sequencing facilities. Processing of the resulting sequences, that is, trimming and quality control, was performed with the MOTHUR software (v 1.30) (Schloss et al. 2009) including denoising of the flowgrams using PyroNoise (Quince et al. 2009) and data normalization or downsampling to the number of sequences in the smallest group. Sequences with ≥250 bp and no ambiguous or no homopolymers ≥8 bp were included for further analysis. These sequences were aligned using the SILVA SSU database (release 108, Pruesse et al. 2007). All sequences were binned into operational taxonomic units (OTUs) and were clustered (average neighbor algorithm) at 97% sequence identity (Stackebrandt and Goebel 1994; Kunin et al. 2010). Coverage values were calculated with MOTHUR (v 1.30). The batch of sequences from this study has been submitted to the Short Reads Archive (http://www.ncbi.nlm.nih.gov/sra) with BioProject ID PRJNA23598.

### Diversity and similarity analysis

The indices of Shannon–Wiener (*H*) (Shannon and Weaver 1949) and Simpson (*D*) (Simpson 1949) were used for diversity estimates. Cluster analysis was applied to Simpson and Morisita similarity with 1000 bootstrap values. Analysis of similarities (ANOSIM) was performed between the phylotype frequencies of the potential groups. All the above analyses were performed using the PAST software (Hammer et al. 2001).

### NMDS analysis

Unconstrained ordinations, based on the frequencies of the phylotypes, were performed in order to graphically illustrate the relationships between different individuals by using two-dimensional nonmetric multidimensional scaling (NMDS) (Kruskal 1964), implemented in *R* (version 2.9.1). NMDS ordination attempts to place all the samples in a two-dimensional space such that their ordering relationships (here based on a Bray-Curtis similarity matrix) are preserved. Hence, the closer the samples are in the resulting ordination, the more similar the bacterial communities are. The Kruskal's stress value reflects the difficulty involved in fitting the relationships of the samples into a two-dimensional ordination space. Fitting of morphometrical vectors was performed using the function envfit in *R* (2.9.1), where squared correlation coefficient values are calculated between NMDS results and morphometric data. The hypothesis that gut microbial communities differ depending on the treatment was tested with the use of the nonparametric ANOSIM (Clarke and Green 1988). ANOSIM generates a test statistic, *R*, that ranges from −1 to 1. The magnitude of R is indicative of the degree of separation between groups, with a score of 1 indicating complete separation and 0 indicating no separation (Clarke 1993).

## Results

### Bacteria

**Figure 1:**
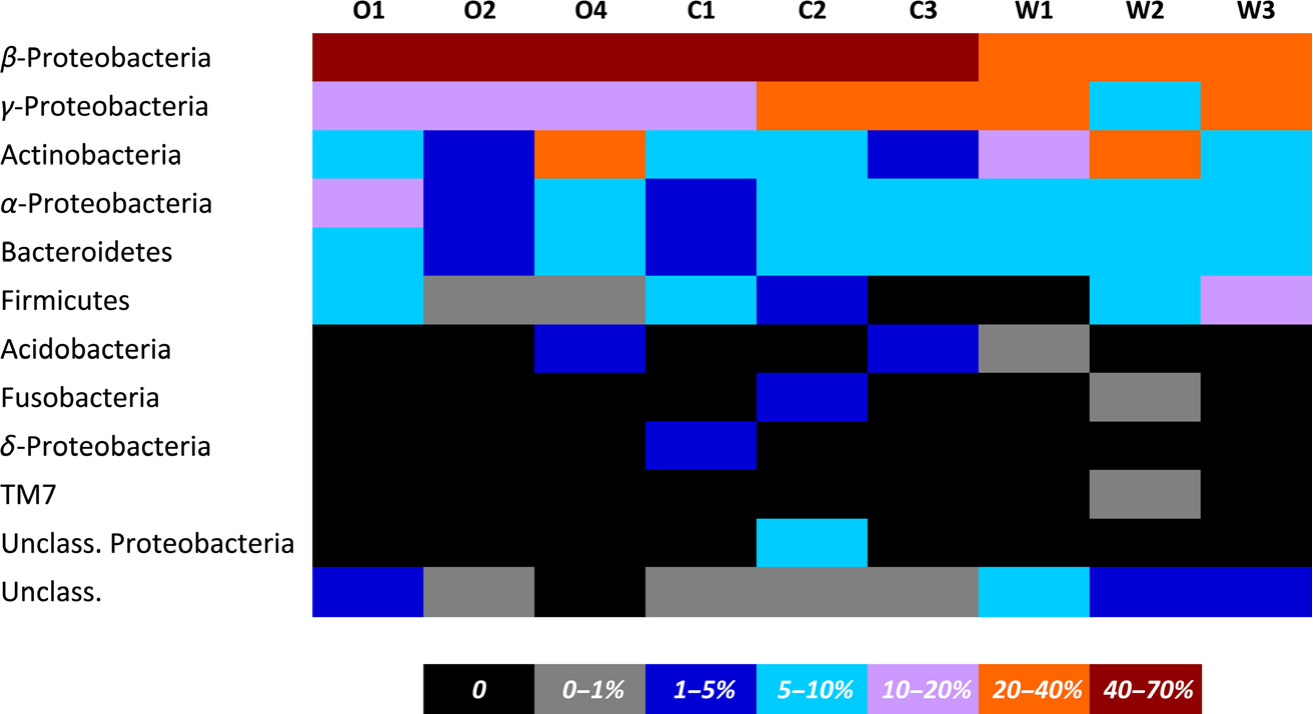
Major bacterial groups found in the gut of wild (W), organically- (O) and conventionally reared (C) sea bream individuals.

In total, 304 unique bacteria OTUs were found in all treatments. After removing single singletons, 196 unique OTUs remained and the number of unique OTUs/sample ranged from 15 (C3) to 53 (W1) (Table2). The majority of the detected OTUs clustered in Actinobacteria (23.6%), followed by Firmicutes (22.9%), *α*-Proteobacteria (20.6%), *γ*-Proteobacteria (19.8%), Bacteroidetes (19.1%), and *β*-Proteobacteria (16.7%). The rest of the OTUs clustered in Fusobacteria, *δ*-Proteobacteria, Acidobacteria or remained unaffiliated. Regarding OTUs relative abundances, the dominant group in all individuals were the *β*-Proteobacteria, exceeding 30% in all samples, followed, in all but two samples (O4, W2), by the *γ*-Proteobacteria. The second most abundant group in O4 and W2 was the Actinobacteria (Fig.1).

**Table 2:** Relative abundance of the operational taxonomic units (OTU), coverage, and diversity indices of organically reared (O), conventionally reared (C), and wild (W) sea bream (*Sparus aurata*) individuals.

Sample	OTUs/sample	Seqs/sample	Rarefaction	Shannon	Simpson
O1	51	4891	0.99	2.60	0.84
O2	38	12,858	0.99	1.53	0.61
O4	45	9895	0.99	2.52	0.83
C1	34	8254	0.99	2.19	0.77
C2	34	6757	0.99	2.48	0.85
C3	15	248	0.98	1.97	0.78
W1	53	5035	0.99	2.86	0.89
W2	43	2870	0.99	2.51	0.85
W3	45	10,940	0.99	2.48	0.84

**Figure 2:**
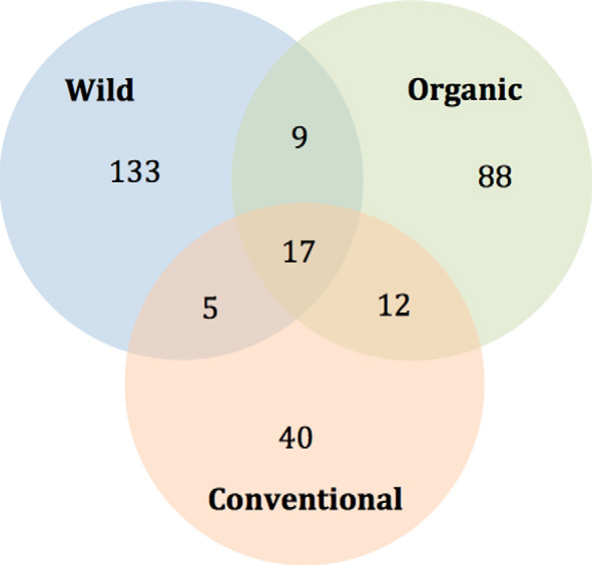
Venn diagram of the average number of shared and unique operational taxonomic units of Bacteria in wild, biologically-, and conventionally reared Sparus aurata gut communities.

Seventeen OTUs were shared among all treatments, while another 26 were shared between two of the treatments, with the highest number (12) being shared between the organic and conventional individuals and the lowest (five) between the wild and the conventional ones (Fig.2). A total of 131 OTUs occurred only in one of the three treatments. The wild, organic, and conventional sea bream hosted 79, 48, and 25 unique OTUs, respectively.

**Figure 3:**
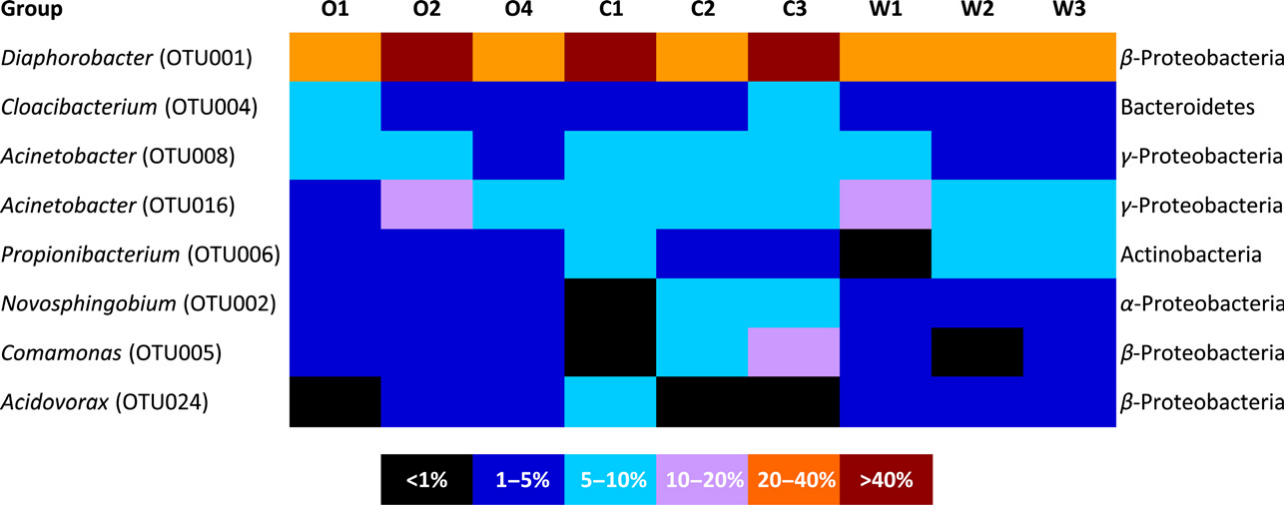
Relative abundance of the most abundant bacterial operational taxonomic units (OTU) found in the gut of wild (W), organically- (O) and conventionally reared (C) sea bream individuals.

At the individual level, only seven OTUs were shared between all nine samples. Among these, four OTUs were present in all samples in frequencies higher than 1%. The dominant OTU in all cases was OTU001, with frequencies ranging from 26.6% (W1) to 60.4 (B2). OTU001 clustered in *β*-Proteobacteria and BLAST search showed that it was closely related with representatives of the genus *Diaphorobacter*. The other three OTUs were OTU004, clustering in the genus *Cloacibacterium* (Bacteroidetes) and OTU008 and 016, clustered in the genus *Acinetobacter* (*γ*-Proteobacteria) (Fig.3, Table S1).

Apart from these four OTUs, other ones also appeared occasionally, or in specific treatments. OTU006, which clustered in the genus *Propionibacterium,* was detected in all organic and conventional samples. The *Comamonas*-related OTU005 was detected in all organic samples, while OTU002, closely related to the genus *Novospingobium,* was detected in all biological and wild samples. Finally, OTU024 was detected in all wild individuals and belonged to the genus *Acidovorax* (Fig3, Table S1).

**Figure 4:**
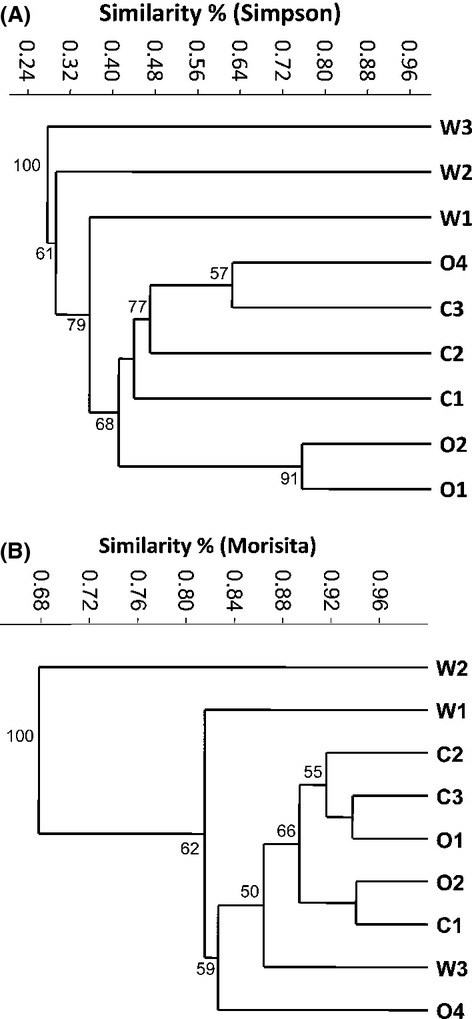
Cluster analysis based on the Simpson and Morisita similarity indices of the bacterial operational taxonomic units (OTU) found in the gut of wild (W), organically- (O) and conventionally reared (C) sea bream individuals. One thousand bootstrap analyses were conducted (values of ≥50 are shown).

MANOVA showed that the conventional sea bream gut bacterial communities were significantly different from those of wild (*P* = 0.007) and organic (*P* = 0.020) ones. In terms of OTU presence/absence, the ratio of shared to total OTU richness in each treatment was 6.1%, 11.1%, and 10.8% for the wild, biological, and conventional sea bream (Fig. S2). The three treatments were distinctively clustered based on the Simpson similarity (Fig.4A). Based on the cluster, the wild sea bream group was less than ca. 35% similar to the rest. With the exception of one organic (O4) and one conventional (C3) sample that were ca. 62% similar, the organic samples were clustered separately from the conventional ones. Cluster analysis with Morisita similarity index (weighted index) had different results since it showed higher similarities, than Simpson index, between samples with the lowest value being 68% (Fig.4B). Diversity indices were overall higher in the wild and organic-reared sea breams with the exception of individual Ο2 that exhibited high dominance and was comparable to the conventional individuals (Table2).

**Figure 5:**
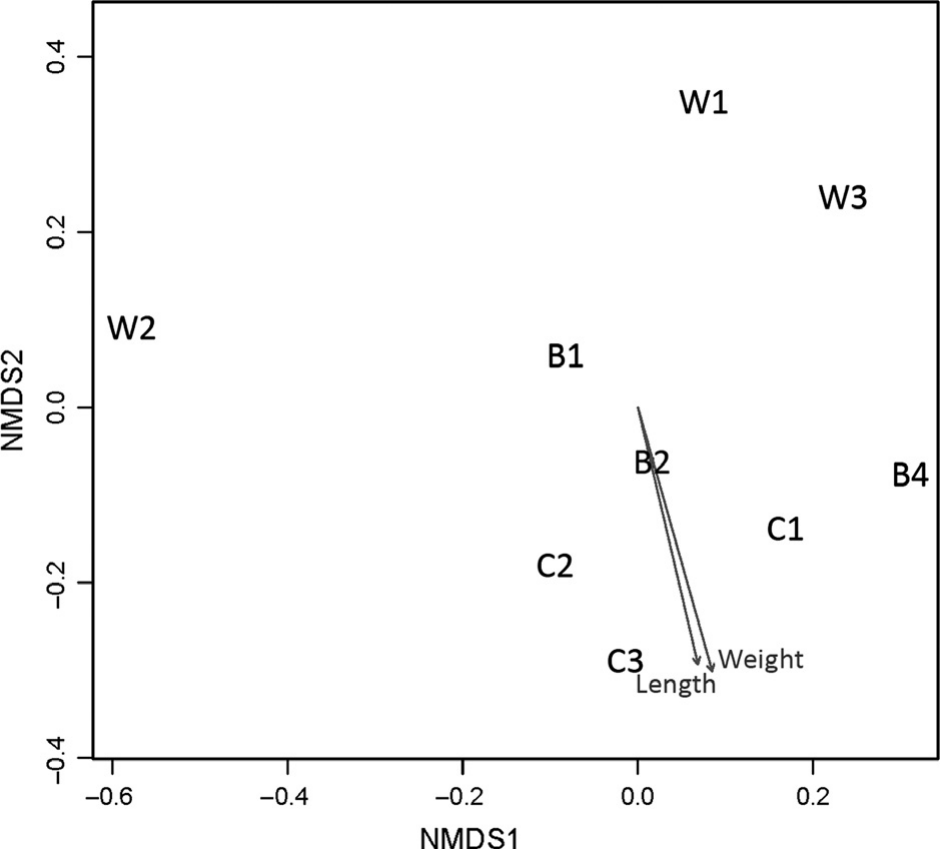
Nonparametric multidimensional scaling (NMDS) ordination plot (Bray-Curtis similarity index) of the bacterial operational taxonomic units (OTU) found in the gut of wild (W), organically- (O) and conventionally reared (C) sea bream individuals.

NMDS analysis (stress: 10.6%) did not exhibit any specific grouping of the samples according to treatment (Fig.5) and this was verified by ANOSIM that exhibited no significance of treatment to the ordination of the samples. On the contrary, the envfit function showed the significance of morphometric factors such as length (*P* = 0.008) and weight (*P* = 0.030) for the ordination of the samples, while liver weight and HIS had no significance.

### Archaea

**Figure 6:**
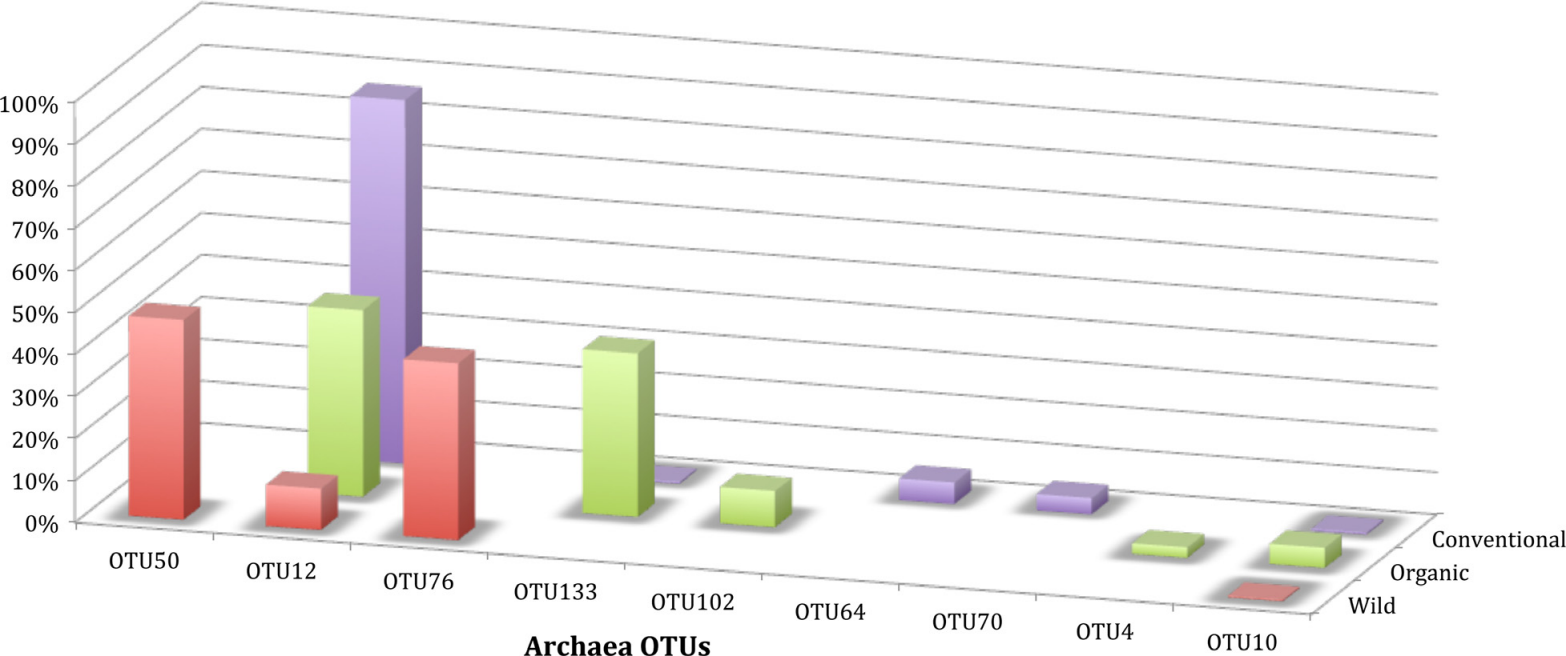
Dominant (cumulative up to 90%) of archaeal operational taxonomic units (OTU) in the gut of wild (W), organically- (O) and conventionally reared (C) sea bream individuals.

In total 27 Archaea OTUs were retrieved (Table1). The highest number of OTUs was found in the organic sea bream (14), followed by the conventional (10) and the wild (9). Although each sea bream was dominated by different OTUs (Fig.6), the majority of the OTUs (20/27) were affiliated to anaerobic methanotrophs and this was more evident amongst the dominant OTUs (Table S2).

## Discussion

This study analyzed gut microbial communities in wild, conventional, and organic sea bream (*S. aurata*) individuals, in order to investigate differences in gut microbial communities and detect frequently co-occurring microorganisms across the host's gut. For the first time next-generation sequencing (NGS) techniques were used for the study of the gut microbiome of the sea bream (*S. aurata*) that had only been studied with the use of conventional techniques (Floris et al. 2013).

MANOVA analysis on bacterial communities results, showed the significant differentiation of conventional-reared samples in comparison to the organic and wild ones. No significant differentiation was detected between organic and wild individuals, suggesting that organic rearing simulates natural conditions better than conventional rearing, in terms of influencing GIT bacterial diversity. Similar results arose from unweighted cluster analysis with Simpson similarity index taking into account similar bacterial OTUs between samples and showing a separate clustering of conventional samples with the exception of one organic-reared individual. However, weighted Morisita similarity index, which is highly sensitive to the frequencies of the most abundant bacterial species in a sample, showed high similarities between samples, exceeding 80%, with the exception of one wild sample (68%). The OTUs which occurred only in one treatment had low relative abundance (0.6–6.4%) suggesting their marginal role at least in terms of dominance in the examined samples.

The dominant phylum, in all individuals, was the Proteobacteria, appearing in frequencies higher than 50% in each sample, but this was due to the dominance of a single OTU, related to *Diaphorobacter* sp. A similar prevalence of Proteobacteria, in the gut microbiome of marine fishes, was reported by Sullam et al. (2012) based on a meta-analysis of previously published data. In the same report (Sullam et al. 2012), the majority of the Proteobacteria analyzed were found to belong to the *γ*-Proteobacteria and more specifically, in the case of carnivorous marine fishes, to the orders Vibrionales and Alteromonadales. Similarly, previous studies in marine fish have shown the predominance of genera such as *Pseudomonas, Acinetobacter, Alteromonas, Aeromonas, Moraxella*, and *Vibrio* in gut bacterial communities (Gómez and Balcázar 2008; Nayak 2010). It has been suggested that the dominance of *γ*-Proteobacteria and Firmicutes is related to diets including plant ingredients compared to fish meal diets (Mansfield et al. 2010; Desai et al. 2012). However, in this study, in all sea bream individuals, the majority of bacteria detected clustered in *β*-Proteobacteria.

This is the first time that such a prevalence of *β*-Proteobacteria is observed in the gut microbiome of a marine fish, while the usually predominant bacteria, such as members of Pseudomonadales, Vibrionales, and Alteromonadales were only detected in lower frequencies (Fig. S3). A possible factor for these differences, may be the material used for the microbial community analysis in these studies and some methodological limitations, like differential DNA extraction or primer specificity of some bacterial groups. Sea bream gut microbial community was studied by analyzing the gut tissue, focusing in more likely resident than transient microbiota, while the majority of previous studies as reported in Sullam et al. (2012) had used intestinal contents or feces.

The only available study to date which analyzed the gut microbiome of sea bream, involved only conventional bacterial counts methods and subsequent genetic analysis (Floris et al. 2013). The dominant species detected in this case was *Pseudomonas* spp., but the result is not comparable to our study since the methods used have a different resolution with NGS techniques, the latter being able to detect a much broader range of microorganisms than conventional microbiology techniques.

In our study we have found four OTUs which occurred across all investigated subjects at similar relative abundances. This is of particular importance for the three wild individuals which had no close contact and most likely had been feeding in great distances between them. The dominant OTU in all individuals, irrespective of treatment, was OTU001 belonging to the *β*-Proteobacteria, a phylum being more ubiquitous in freshwater habitats (Barberán and Casamayor 2010). OTU001 was closely related to the genus *Diaphorobacter,* a denitrifier known to degrade polyaromatic hydrocarbons via denitrification (Klankeo et al. 2009). Based on the osmoregulation of gut cells in seawater-adapted fish (Taylor et al. 2011), their nonsaline habitat could favor *Diaphorobacter* spp. growth, which takes place in media with no NaCl (Khan and Hiraishi 2002; Pham et al. 2009). The other three OTUs appearing in all samples clustered in the genera *Cloacibacterium* and *Acinetobacter*. The recently described species *Cloacibacterium haliotis* has been isolated from the abalone *Haliotis discus hannai* (Hyun et al. 2014). *Acinetobacter* spp. can metabolize several organic compounds such as amino acids, aromatic compounds, short-chain fatty acids (Towner 2006) and could be likely contributing to the animal's nitrogen and carbohydrates metabolism. Finally, a total of 23.6% of the found OTUs belonged to the Actinobacteria, a cosmopolitan phylum in freshwater environments, just like the *β*-Proteobacteria (Barberán and Casamayor 2010; Newton et al. 2011).

The presence of common ‘resident’ OTUs in all gut bacterial communities, with relative abundances/individual exceeding 35% suggests the presence of a bacterial community in the sea bream gut with potential role in the nutrition and/or the immunity of the host. This study used molecular tools for the analysis of 16S rRNA, so all the interpretations for the processes that might occur in the gut have to be made with great caution, since no cultivations and biochemical tests were performed. Thus, the physiology of closest relatives can only give hints about the potential metabolic pathways that are realized. Studying the physiology of the closest relatives of the genera detected in this study, it is implied that biochemical processes such as denitrification, fermentation, and degradation of aromatic compounds could occur in the gut of *S. aurata*. Apart from *Diaphorobacter* sp., other species closely related to dominant OTUs (Fig. S1) such as *Novosphingobium, Comamonas*, and *Acidovorax* are able for nitrate reduction (Dworkin et al. 2006), potentially assisting in the metabolism of nitrogenous compounds. Fermentation performed by *Cloacibacterium* sp., is a major process for the metabolism of glucose in short-chain fatty acids that might be used later in other chemoautotrophic processes. Finally the degradation of organic molecules such as amino acids, polyaromatic hydrocarbons, and aromatic compounds that is performed by strains of *Acinetobacter, Diaphorobacter*, and *Comamonas* could be important for the metabolism of complex and potentially toxic organic molecules.

Regarding Archaea, amplifiable DNA was detected only for one individual per treatment, implying that Archaea are considerably underrepresented in the sea bream gut as is the case for other similar microbial habitats (Smeti et al. 2013). The majority of the Archaea detected in this study clustered in the Euryarchaeota, class of Methanomicrobia. Members of this family are associated with methanogenesis and/or anaerobic oxidation of methane. Members of the Methanomicrobia, clustered in the genus *Methanococcoides* have been previously detected in the intestinal contents and the feces of fish caught in the North Sea (van der Maarel et al. 1999), but their exact function and origin remains elusive. It has been suggested that the occurrence of methanogens is possible in anaerobic niches of gut habitats, where methanogens can be hosted as commensals participating in nutrient decomposition (Wrede et al. 2012), as is the case for *Methanobrevibacter smithii* which dominates the archaeal assemblage of the human gut (Eckburg et al. 2005).

Although archaeal diversity was not as well examined as bacterial diversity since only one sample per treatment was analyzed, the presence of anaerobic methanotrophs in very high relative abundances exceeding 57%, implies the significance of archaeal communities in the gut of *S. aurata*. This finding, however, allows only hypotheses to be made for the functions of archaea in the gut and for the interactions between archaeal and bacterial communities. It is striking though, that representatives of the most abundant genus *Diaphorobacter* sp., have been detected in coal beds supporting methanogenesis by members of Methanobacteria via denitrification (Singh et al. 2012).

In conclusion, this study revealed that the different feeding mode reflects on the gut prokaryotic communities structure in conventional-reared, organic-reared, and wild *S. aurata* individuals. However, a few OTUs were found to dominate in all individuals, implying the existence of a bacterial community with unknown function. Based on the physiology of the most abundant and frequently found OTUs’ closest relatives, denitrification, fermentation, and degradation of complex organic compounds arose as potentially important processes that take place in the gut. The decreasing numbers of gut OTUs from the wild to organic and then to conventional sea bream, might indicate a less functional nutritional status or it could related to the more restricted diversity of food items of the reared sea breams compared to the wild ones. For example, low number of bacterial OTUs has been associated with fatty diet of obese humans (Le Chatelier et al. 2013). In order to clarify this, along with the prevailing metabolic pathways related to fish nutrition, further research is required on the metabolic capacities of these GIT microorganisms.

## Supporting Information

**Figure S1.** Rarefaction curves of the wild (W), organically- (O), and conventionally (C) reared *Sparus aurata* (Sa) gut bacterial communities. OTUs: operational taxonomic units. **Figure S2**. Number of shared operational taxonomic units among the triplicate samples of the wild (W), organically- (O), and conventionally (C) reared *Sparus aurata* (Sa) gut bacterial communities. **Figure S3**. Heatmap of the most abundant operational taxonomic units found in the gut of wild (W), organically- (O), and conventionally reared (C) sea bream individuals. **Table S1**. Dominant (>90%) Bacteria operational taxonomic units (OTU) in the gut of wild (W), biological (b), and conventional (C) sea bream. **Table S2**. Dominant (>90%) Archaea operational taxonomic units (OTU) in the gut of wild (W), biological (b), and conventional (C) sea bream.
