# Supplementary material for: Dietary differences are reflected on the gut prokaryotic community structure of wild and commercially reared sea bream (Sparus aurata)
Source: Microbiologyopen. 2014 Jul 25;3(5):718–28. doi: 10.1002/mbo3.202 (PMC4234263; doi:10.1002/mbo3.202)
Supplement: Supplementary file 1 — Figure S1. Rarefaction curves of the wild (W), organically- (O), and conventionally (C) reared Sparus aurata (Sa) gut bacterial communities. OTUs: operational taxonomic units. Figure S2. Number of shared operational taxonomic units among the triplicate samples of the wild (W), organically- (O), and conventionally (C) reared Sparus aurata (Sa) gut bacterial communities. Figure S3. Heatmap of the most abundant operational taxonomic units found in the gut of wild (W), organically- (O), and conventionally reared (C) sea bream individuals. Table S1. Dominant (>90%) Bacteria operational taxonomic units (OTU) in the gut of wild (W), biological (b), and conventional (C) sea bream. Table S2. Dominant (>90%) Archaea operational taxonomic units (OTU) in the gut of wild (W), biological (b), and conventional (C) sea bream. [file mbo30003-0718-sd1.doc]

**Dietary differences are reflected on the gut prokaryotic community structure of wild and commercially reared sea bream (*Sparus aurata*)**

Konstantinos A. Kormas^1^*, Alexandra Meziti^1^, Eleni Mente^1^, Athanasios Frentzos^2^

^1^ Department of Ichthyology & Aquatic Environment, School of Agricultural Environment, University of Thessaly, 384 46 Volos, Greece

^2^ Kefalonia Fisheries, Livadi, Lixouri Kefalonia 28200, Greece

* Corresponding author; Tel.: +30-242-109-3082, Fax: +30-242-109-3157, E-mail: kkormas@uth.gr

**Supplementary material**

**Submitted to “MicrobiologyOpen”**


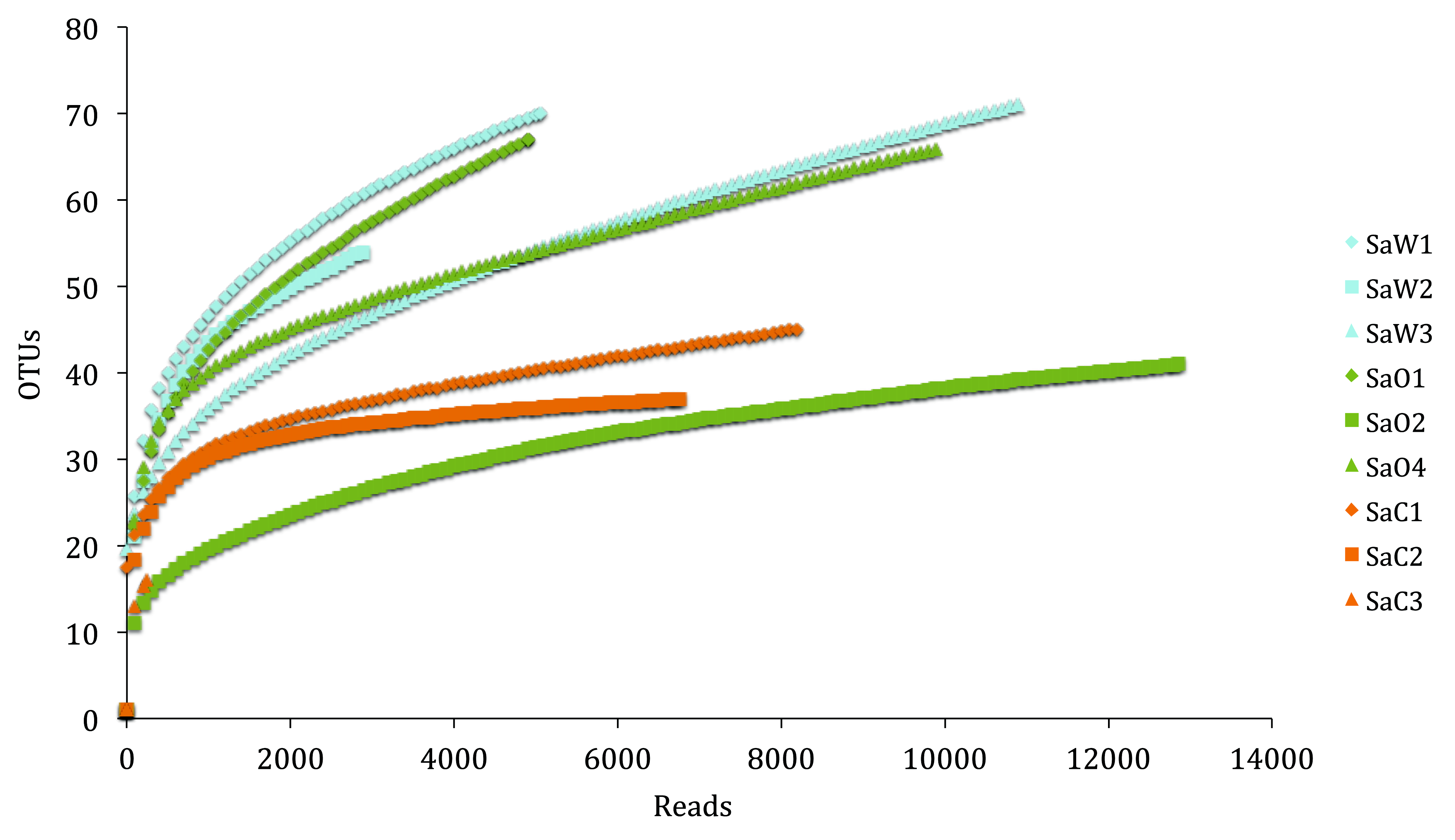


**Figure S1**. Rarefaction curves of the wild (W), organically (O) and conventionally (C) reared *Sparus aurata* (Sa) gut bacterial communities. OTUs: operational taxonomic units.


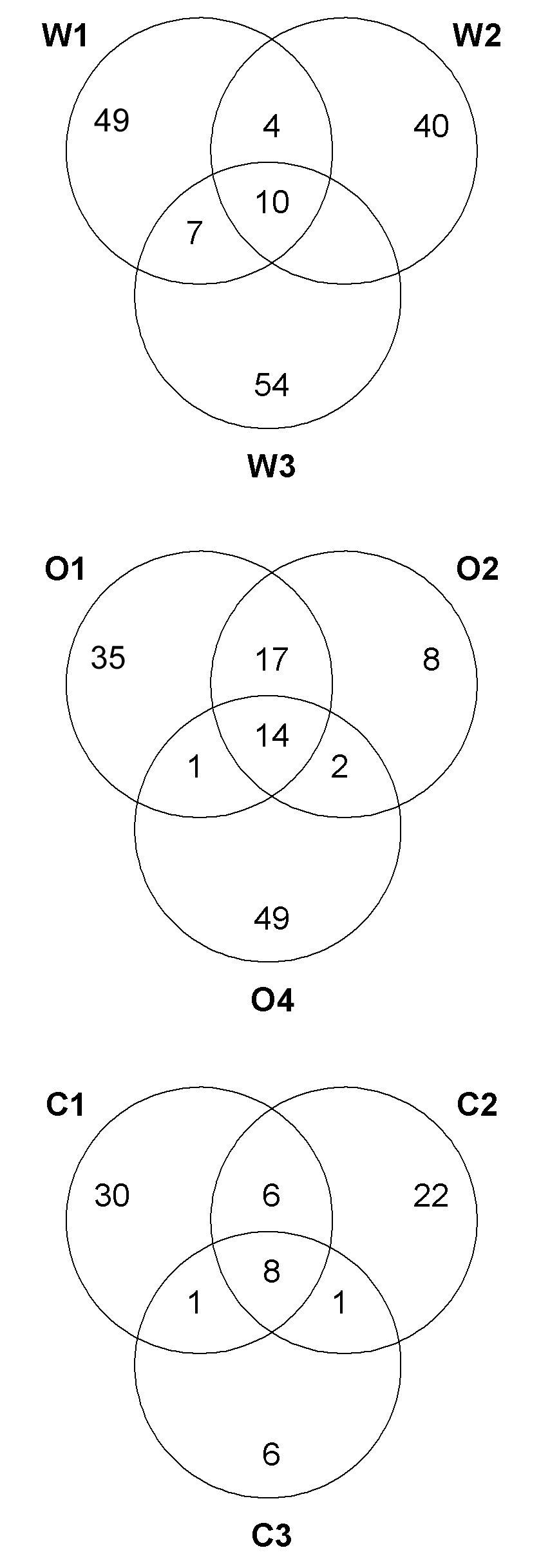


**Figure S2**. Number of shared operational taxonomic units among the triplicate samples of the wild (W), organically (O) and conventionally (C) reared *Sparus aurata* (Sa) gut bacterial communities.

**

**

**Figure S3**. Heatmap of the most abundant operational taxonomic units found in the gut of wild (W), organically- (O) and conventionally-reared (C) sea bream individuals.

**Table S1**. Dominant (>90%) Bacteria operational taxonomic units (OTU) in the gut of wild (W), biological (b) and conventional (C) sea bream.

| **OTU** | **Average %**  **(N=3)** | | | **Putative affiliation**  **(phylum)** | **Closest (>97%) relative [GenBank accession No.]** | **Description** | **Reference** |
| --- | --- | --- | --- | --- | --- | --- | --- |
|  | W | B | C |  |  |  |  |
| 001 | 31.6 | 47.1 | 39.9 | β-Proteobacteria | *Diaphorobacter* sp. DS3 [JX272921] | Industrial effluent | Unpublished |
| 002 | 2.1 | 3.2 | 3.8 | Unaffiliated | Clone MID27_4188 [JX107951] | Cow uterus | Santos & Bicalho (2012) |
| 003 | 0.9 | 1.3 | 1.5 | α-Proteobacteria | *Sphingobium* sp. 6005 [JX566637] | Purple soil | Unpublished |
| 004 | 2.4 | 3.1 | 3.4 | Unaffiliated | Clone G8XQE6O04JVRXA [JX635391] | Mastitic milk | Oikonomou et al. (2012) |
| 005 | 1.6 | 3.5 | 3.0 | β-Proteobacteria | *Comamonas* sp. KB20 [KC295208] | Swine sludge | Unpublished |
| 006 | 4.8 | 3.2 | 4.7 | Unaffiliated | Clone F1Q32TO06G8H4M [GU518723] | Wastewater | Kwon et al. (2010) |
| 007 | 0.0 | 0.9 | 0.0 | β-Proteobacteria | *Achromobacter arsenitoxydans* strain BR2_14 [KC461205] | rhizosphere | Unpublished |
| 008 | 3.2 | 5.0 | 8.1 | Unaffiliated | Clone Pd4H16 [JX644373] | Earthworm nephridia | Davidson et al. (2013) |
| 009 | 0.0 | 0.6 | 0.0 | Firmicutes | Clone CF1-42 [GU958919] | Rat faeces | Unpublished |
| 015 | 0.0 | 0.0 | 1.3 | Unaffiliated | Clone SB-48 [KC470439] | high Arctic subzero hypersaline spring | Unpublished |
| 016 | 9.2 | 8.1 | 7.6 | γ-Proteobacteria | *Acinetobacter junii* E91 [HF585196] | Endophyte | Unpublished |
| 024 | 2.4 | 2.4 | 3.0 | Unaffiliated | Clone MID27_428 [JX106752] | Cow uterus | Santos & Bicalho (2012) |

References

Davidson,S.K., Powell,R. and James,S. (2013) A global survey of the bacteria within earthworm nephridia. Mol. Phylogenet. Evol. 67 (1), 188-200

Kwon,S., Kim,T.S., Yu,G.H., Jung,J.H. and Park,H.D. (2010) Bacterial community composition and diversity of a full-scale integrated fixed-film activated sludge system as investigated by pyrosequencing. J. Microbiol. Biotechnol. 20 (12), 1717-1723.

Oikonomou,G., Machado,V.S., Santisteban,C., Schukken,Y.H. and Bicalho,R.C. (2012) Microbial diversity of bovine mastitic milk as described by pyrosequencing of metagenomic 16s rDNA. PLoS ONE 7 (10), E47671

Santos,T.M. and Bicalho,R.C. (2012) Diversity and succession of bacterial communities in the uterine fluid of postpartum metritic, endometritic and healthy dairy cows. PLoS ONE 7 (12), E53048

**Table S2**. Dominant (>90%) Archaea operational taxonomic units (OTU) in the gut of wild (W), biological (b) and conventional (C) sea bream.

| **OTU** | **%** | | | **Putative affiliation**  **(phylum)** | **Closest (>93%) relative [GenBank accession No.]** | **Description** | **Reference** |
| --- | --- | --- | --- | --- | --- | --- | --- |
|  | W | B | C |  |  |  |  |
| 50 | 47.4 | 0.0 | 0.0 | Euryarchaeota  (ANME-1) | Clone fos0128g3+03e1 [CR937008] | methane rich Black Sea microbial mat | Meyerdierks et al. (2005) |
| 12 | 9.8 | 44.3 | 86.4 | Euryarchaeota  (ANME-2c) | Clone NZ_309_Arch50 [JQ241737] | Deep-sea methane cold seep | Unpublished |
| 76 | 42.0 | 0.0 | 0.0 | Thermoplasmata  (DHVEG-1) | Clone PM-152-75-M13R [JQ925198] | Marine methane cold seep | Unpublished |
| 133 | 0.0 | 38.9 | 0.4 | Euryarchaeota  (ANME-2b) | Clone AMSMV-20-A32 [HQ588672] | Deep-sea methane mud volcano | Pachiadaki et al. (2011) |
| 102 | 0.0 | 8.6 | 0.0 | Euryarchaeota  (Methanosarcinales) | Clone BCa2c_ar1F3 [EU622308] | Deep-sea methane cold seep | Pernthaler et al. (2008) |
| 64 | 0.0 | 0.0 | 5.1 | Euryarchaeota (MG II) | Clone ST010515arc1aG4 [JX281656] | Coastal seawater | Unpublished |
| 70 | 0.0 | 0.0 | 3.8 | Thaumarchaeota | Clone LF_16SA_UIF_D-E8 [JX015356] | Saline lake interface | Unpublished |
| 4 | 0.0 | 2.5 | 0.0 | Euryarchaeota | Clone NZ_315_Arch_60 [JQ241757] | Deep-sea methane cold seep | Unpublished |
| 10 | 0.3 | 4.8 | 0.4 | Euryarchaeota  (ANME-3) | Clone KZNMV-30-A1 [FJ712392] | Deep-sea methane mud volcano | Pachiadaki et al. (2010) |

References

Meyerdierks,A., Kube,M., Lombardot,T., Knittel,K., Bauer,M., Glockner,F.O., Reinhardt,R., Amann,R. (2005) Insights into the genomes of archaea mediating the anaerobic oxidation of methane. Environ. Microbiol. 7 (12), 1937-1951

Pachiadaki,M.G., Kallionaki,A., Dahlmann,A., De Lange,G.J., Kormas,K.A. (2011) Diversity and spatial distribution of prokaryotic communities along a sediment vertical profile of a deep-sea mud volcano. Microb. Ecol. 62 (3), 655-668.

Pachiadaki,M.G., Lykousis,V., Stefanou,E.G., Kormas,K.A. (2011) Prokaryotic community structure and diversity in the sediments of an active submarine mud volcano (Kazan mud volcano, East Mediterranean Sea). FEMS Microbiol. Ecol. 72 (3), 429-444.

Pernthaler,A., Dekas,A.E., Brown,C.T., Goffredi,S.K., Embaye,T., Orphan,V.J. (2008) Diverse syntrophic partnerships from deep-sea methane vents revealed by direct cell capture and metagenomics. Proc. Natl. Acad. Sci. U.S.A. 105 (19), 7052-7057.
